# Supplementary material for: Lessons from 20 years of medical cannabis use in Canada
Source: PLoS One. 2023 Mar 23;18(3):e0271079. doi: 10.1371/journal.pone.0271079 (PMC10035846; doi:10.1371/journal.pone.0271079)
Supplement: Attachment — Submitted filename: plos_one_review.pdf [file pone.0271079.s001.pdf]

Overview and Recommendation:

The authors provide an extensive descriptive history of the Canadian medical cannabis regime by collating data from a wide variety of sources to provide an integrated overview of how the sector has evolved since inception. An extensive amount of data is processed to provide statistical snapshots of several dimensions of the Canadian medical cannabis system over time, including after legalization. The analysis I believe is mostly novel, the manuscript is well-written if not always technically clear, and there are no statistical errors that I can identify.

Unfortunately I can only recommend that PLOS reject the manuscript. The data handling, transparency, and accessibility clearly do not meet the standards of PLOS. This could be potentially fixed, which makes a case for major revisions, but my reading of the manuscript and the authors' declarations suggests they will be unable to meet the PLOS data standards and hence I recommend rejection. I lay out a number of crucial issues leading to this recommendation below.

Key Issues:

My reading of the PLOS data policy, as provided to reviewers, holds data work to a very high standard: "requires authors to make all data underlying the findings described in their manuscript fully available without restriction, with rare exception…[t]he data should be provided as part of the manuscript or its supporting information, or deposited to a public repository".

The authors clearly do not meet this standard, nor do they even acknowledge it or the scientific goals of PLOS. The authors' response to the "Data Availability" statement simply says "No – some restrictions will apply." The authors make no attempt to describe what restrictions will apply, or what data sources will be made available. I cannot tell whether 90% or 20% of the data will be made available. No data was uploaded for reviewers to examine, and they provide no argument at all why they should be exempt from the data availability policy. This is the antithesis of what PLOS is trying to achieve.

The promised use of Google Drive to share the limited data that will be made available does not, from my reading, conform to the data policy either. Google Drive accounts are individual accounts, not public repositories. Uploading to Google Drive means that future researchers cannot rely on accessing the data that will be provided.

Absent access to the data, much of the data is not sufficiently well described to be fully understood. For example, the authors collect data by "monitor[ing] the on-line product catalogs of Licensed Producers each month from June 2016". There are hundreds and hundreds of LPs. How were these websites monitored? Is this a complete sample of LPs or a highly selected sample? This is potentially a massive data collection effort that the reader cannot fully understand from the text and may not be provided access to, making it impossible to replicate, to build on, or to examine the subtleties of. The blanket references to unsourceable private administrative data from Health Canada's Office of Medical Cannabis are even more opaque.

The methods section describes a lot of sources, but then the reader struggles to link statistics back to the actual source, especially when these claims are at times extreme. For example, the authors claim the average daily dose of authorized cannabis was 20.6 grams/day in 2013. This is wildly inconsistent with the existing literature that I'm aware of. Unfortunately, I cannot link this statement back to any particular source. The reader is told of many data sources, and then told of many results coming from

these sources, but is often not given consistent insight on where certain numbers come from, preventing interpretation of the results.

I agree there is a potentially great resource here if the authors could collate and share 100% of the data from their work to the scientific community, but my reading suggests there is no intention to do so. The work would be much better suited for an outlet focused more on results and less on transparency and process. Alternatively, there is probably enough here for two papers if the authors wanted to split the paper and fully develop the results – perhaps one with the data they can share and one with the data they are unwilling or unable to.

<u>Detailed Comments:</u>

Fig 2 is an important graph that doesn't exist this starkly anywhere else in the literature to the best of my knowledge. The source is given as "Health Canada Office of Medical Cannabis", but the "Office of Medical Cannabis" doesn't appear in the data section, and I can't find any website belonging to the OMC that indicates what data might or might not be available? Some discussion of how this data was/is/could be obtained is required.

The reference [17] attached to the discussion of Figure 2 does not actually provide useful context for the discussion, it just links to a list of LPs but this list seems to be continually updated to reflect changing market conditions and cannot reflect a static list of LPs that the authors actually surveyed in the past.

Fig 3 is interesting but see earlier comments. Is this using some sort of archived website scraping? Is this a complete sample of LPs? Is this a simple average or weighted by LP size? I don't think the biggest LP should carry the same weight in this calculation as the 200th-biggest LP.

The literature should be acknowledged here. My understanding is that other studies that have looked at what's being sold medically find very different things. In particular the finding that a THC-dominant oil held a commanding market share until 2021 is not what I have read elsewhere. Indeed only 6 of the references are peer-reviewed studies on cannabis. I believe most of what's being done is novel but there's limited effort by the authors to place their findings in the literature.

Fig 4 again interesting, but this is maybe not novel. Statscan maintains good data on prices, probably better data than is presented here. Fig 4 also claims to be a "mean", but this is unclear. The data source is given as monthly review of catalogs, but this isn't a mean because the catalog review doesn't tell you volumes sold, it's an average across product lines not by amount sold, and the latter seems to be what we actually want.

I think maybe the measurement of oils needs some disclaimers. mL is only relevant if different LPs compound their products at roughly the same % active ingredient, but my understanding is there are significant differences between LPs in the strength of their formulations, so $/mL is possibly not a meaningful statement.

The discussion/conclusion section doesn't clearly make the reader aware of what is novel.
