# Supplementary material for: Lessons from 20 years of medical cannabis use in Canada
Source: PLoS One. 2023 Mar 23;18(3):e0271079. doi: 10.1371/journal.pone.0271079 (PMC10035846; doi:10.1371/journal.pone.0271079)
Supplement: Attachment — Submitted filename: Response to PLOS Reviews.docx [file pone.0271079.s002.docx]

We thank the referees for their conscientious reviews of our paper. Below, we outline how we have responded to their comments in the revised version of the paper.

Reviewer #1: This is a potentially interesting and useful paper on the evolution of Canada’s medical cannabis system during the past two decades. The paper points to an interesting story about the interplay of changes in policy at the federal and provincial levels and how those change were reflected in amount of cannabis produced and consumed, the nature of cannabis products, and price of cannabis. The paper would be improved by some edits to help make the paper more accessible to a non-Canadian audience and more clearly describing variation and changes provincial policies. Below are specific suggestions.

Comment R1.1. Not being Canadian, I was unsure what Health Canada and physician Colleges are. The authors should make edits in the Abstract and throughout the paper to make the paper more accessible to a non-Canadian audience. I believe that physician Colleges set province-specific guidelines for medical cannabis, but that is not clearly stated anywhere in the paper.

We have now described Health Canada and the Colleges of Physicians and Surgeons in the paper.

Comment R1.2. The interplay between provincial and federal regulations could perhaps be discussed more clearly. Pre-2014, it seems there was a huge difference across provinces in authorizations for medical cannabis (lots more medical cannabis authorized in BC), but this changed in 2014 when a system of commercial growers was created. After 2014, it looks like the size of the medical cannabis market was relatively small in BC. I don’t think this dramatic shift is clearly explained in the paper. Maybe there is no easy explanation. There is a story here about variation in policies, regulations, and maybe enforcement of regulations across provinces and how those things evolved across years.

The paper now has a more fulsome investigation of factors affecting province-specific rates of Licensed Producer (LP) shipments in the MMPR, after personal production (previously allowed under the MMAR) was banned. The paper originally focused on differences across provinces in cannabis prescribing restrictions. But as the referee notes this explanation is insufficient, and we agree. In the revised version, we now identify the possible role of additional factors, notably the impact of the number of personal or delegated cannabis cultivation licenses under the previous MMAR policy and variation in enforcement of laws governing cannabis use outside the confines of the MMPR. We write in the results section:

Fig 7 also indicates that some variation in the volume of Licensed Producer shipments is due to factors other than prescribing restrictions. In particular, British Columbia and Manitoba have particularly low shipment volumes, and Alberta, high volumes, given their prescribing restriction score. The low volume of Licensed Producer shipments to British Columbia and Manitoba could be due to medical cannabis users in these provinces accessing cannabis from plants that were established under production licenses in the previous MMAR regime.

We expand on this in the discussion section

It also seems plausible that the low rate of program participation in British Columbia was due to the availability of cannabis from dispensaries. In less populous regions of the province, in which the federal police force, the Royal Canadian Mounted Police (RCMP), provided local law enforcement, illicit cannabis distribution was forced to go underground. However, law enforcement agencies in some large cities in the province allowed cannabis dispensaries to operate. (28) The City of Vancouver even regulated cannabis dispensaries, despite their illegality at the time. (29) Province-wide, the average penalty issued for cannabis possession in BC was lower than in other provinces. (30) This suggests that some of the interprovincial variation in the participation in the MMPR regime was due to variation in the propensity of authorities to enforce the CDSA.

Comment R1.3. The section spanning pages 4 through 6 could be more succinct and would perhaps be helped by a figure showing a policy-change timeline. Ideally, you might have separate timelines for each province as well, although I am not sure that information on changes in province-level policies across years is available to the authors. (They do have information on provincial variation in restrictions in 2016.)

We have trimmed this section as much as possible. We also now include as Figure 1 the timeline of changes to the federal government medical cannabis access regime. (Medical cannabis access is outside of provincial government jurisdiction so there are no provincial government policy changes to report.) The provincial College restrictions, as far as we are aware, did not change materially after they were introduced.

Comment R1.4. The issue of variation in enforcement of cannabis laws might be worth addressing more explicitly. In the US, one aspect of liberalization of cannabis policy is decriminalization, involving reduced priority given to enforcement of cannabis laws as well as reduction in penalties for violation of cannabis laws. I was unable to figure out how much of this is an issue in Canada’s cannabis laws. One guess I have about what happened in BC is that after cannabis growing by those authorized for medical cannabis was banned, very little effort was put into enforcing that ban and people continued to supply themselves by growing their own cannabis (or having a friend who grew their own) rather than switching to authorized commercial growers. There was probably a lot of diversion occurring in BC, although from Figure 8 it does not look like population-wide prevalence of daily or near daily use was much higher in BC than most other provinces.

As we noted above, there was in fact significant variation across jurisdictions in enforcement of the Controlled Drugs and Substances Act, at least as it pertains to cannabis use and sale outside of the medical cannabis access regime. The Act was enforced by the RCMP, the federal police force, at least in regions in which they provided local law enforcement. For instance, I understand that the RCMP police operating in city of Kelowna, BC enforced the Act. Some municipal police forces, however, appeared to either not enforce or not rigorously enforce laws against the retail sale of cannabis or its use outside of the medical cannabis access regime. Notably, the Vancouver BC police department did not shut down illegal dispensaries. Indeed, in the fall of 2021, illegal dispensaries were still operating in Vancouver.

<https://vancouversun.com/news/local-news/illegal-dispensaries-still-operating-in-vancouver-three-years-after-cannabis-act-became-law>

Illegal dispensaries were also operating in Toronto, both before and after the legalization of recreational cannabis.

<https://www.thestar.com/news/gta/2019/07/24/many-toronto-pot-users-still-prefer-illegal-shops-heres-why.html>

The Toronto Police did, however, shut some dispensaries down.

<https://www.bbc.com/news/world-us-canada-49024678>

Unfortunately, there are many different local law enforcement agencies across Canada and I don’t really know their degree of enforcement of the relevant portions of the Controlled Drugs and Substances Act. I do, however, have access to province-year level Statistics Canada data on criminal code convictions in respect of cannabis possession and distribution. I could attempt to tease out the stringency of enforcement of the CDSA if I could somehow control for the underlying level of illicit cannabis use. The referee has raised an important research question; my sense is that this line of research would need to be pursued in a separate paper given space constraints.

Comment R1.5. There were some numbers that were surprising to me about the average number of grams per day per medical cannabis user. In dry form, a gram of cannabis is about 3 joints. Someone who uses daily and heavily might use 1 to 2 grams per day. I was surprised to see 18 grams per day allowed under MNAR in 2013 (page 5) or the average daily dose of cannabis authorized by Health Canada of 5.6 grams (page 10). Even 2 grams per day prescribed by physicians under MMPR (page 11) seems awfully high. Am I misunderstanding something?

Re the legitimacy of these high daily dose values, we turn to pages 288-292 of the Allard federal trial transcripts. This is online at the URL

https://johnconroy.com/pdf/Allard-Trial-Transcripts-COMPLETE.pdf

This section reveals two reasons why the dosages were so high. First, some individuals authorized to use cannabis under the MMAR suffered from multiple mental and physical health problems. These individuals used a lot of cannabis just to get through their day. Second, the plaintiffs in the Allard trial also suggested, under cross examination, that they vaporized or made juice out of the cannabis buds and that way of ingesting the active ingredients in cannabis requires higher dosages than the amount required if cannabis was smoked.

It also seems very likely that some of the cannabis authorized under the MMAR (especially in BC) was diverted, or stockpiled.

This is bolstered by the fact that under the MMPR/ACMPR, the actual dosages were somewhat less than the physician authorized dosages. For instance, in the fourth quarter of 2016, the mean physician authorized daily dose was 2.5 grams but the average daily amount per Licensed Producer shipment was 0.79 grams. (see https://www.canada.ca/en/health-canada/services/drugs-medication/cannabis/licensed-producers/market-data.html)

Health Canada remains concerned about the large physician-authorized daily doses of cannabis in BC and in some other provinces. They have called attention to the problem in their website. (See “Highlights of the data as of March 2022:” at <https://www.canada.ca/en/health-canada/services/drugs-medication/cannabis/research-data/medical-purpose.html>

Comment R1.6. The authors put information on variation in medical cannabis restrictions by province in 2016 in an online supplement. I would like to see this table included in the body of the paper.

The information on medical cannabis prescribing restrictions, by province, now appears as Table 2 in the body of the paper.

Comment R1.7. In examining national survey data on cannabis use, the authors focus on daily or near daily use. What was the cutoff for daily use? A common cutoff is 20+ days of use in the past 30. Is that what was used here?

The interview guides for the national surveys that we rely on do not provide any hard cutoffs; it thus appears that the respondents with near daily cannabis use were required to use their own cutoffs. This will add some noise to our estimates of the fraction using daily or almost daily.

Comment R1.8. The authors provide information on price and potency separately for dry cannabis and oils. The authors should briefly clarify how these are linked to different routes of administration (e.g., smoking, vaping, dabbing, and edibles).

We now clarify that dry cannabis is normally smoked and sometimes vaped, while the oils are used primarily for vaping.

Comment R1.9. Does Figure 4 correct for inflation?

The prices displayed in Figure 4 are nominal prices, but we agree that using real prices is more appropriate given the length of time spanned by our data series. We therefore adjust the nominal prices using the Statistics Canada all items national consumer price index with 2016-07=100.

Reviewer #2: 

The authors provide an extensive descriptive history of the Canadian medical cannabis regime by collating data from a wide variety of sources to provide an integrated overview of how the sector has evolved since inception. An extensive amount of data is processed to provide statistical snapshots of several dimensions of the Canadian medical cannabis system over time, including after legalization. The analysis I believe is mostly novel, the manuscript is well-written if not always technically clear, and there are no statistical errors that I can identify.

Comment R2.1. Unfortunately I can only recommend that PLOS reject the manuscript. The data handling, transparency, and accessibility clearly do not meet the standards of PLOS. This could be potentially fixed, which makes a case for major revisions, but my reading of the manuscript and the authors’ declarations suggests they will be unable to meet the PLOS data standards and hence I recommend rejection. I lay out a number of crucial issues leading to this recommendation below.

Key Issues:

My reading of the PLOS data policy, as provided to reviewers, holds data work to a very high standard: “requires authors to make all data underlying the findings described in their manuscript fully available without restriction, with rare exception...[t]he data should be provided as part of the manuscript or its supporting information, or deposited to a public repository”.

The authors clearly do not meet this standard, nor do they even acknowledge it or the scientific goals of PLOS. The authors’ response to the “Data Availability” statement simply says “No – some restrictions will apply.” The authors make no attempt to describe what restrictions will apply, or what data sources will be made available. I cannot tell whether 90% or 20% of the data will be made available. No data was uploaded for reviewers to examine, and they provide no argument at all why they should be exempt from the data availability policy. This is the antithesis of what PLOS is trying to achieve.

The promised use of Google Drive to share the limited data that will be made available does not, from my reading, conform to the data policy either. Google Drive accounts are individual accounts, not public repositories. Uploading to Google Drive means that future researchers cannot rely on accessing the data that will be provided.

We will share all data except the individual respondent level data from the population health surveys. Licensing restrictions prevent us from re-publishing these data. We were remiss not to clarify these points earlier. We now make these points clear.

We also agree that it is better to house the data on a dedicated archival website than on a Google drive. Thus, we will share all unrestricted data on ODESI, the Ontario Data Documentation, Extraction Service and Infrastructure server should the manuscript be accepted for publication in PLOS One. The ODESI website URL is

http://odesi2.scholarsportal.info/webview/

Comment R2.2. Absent access to the data, much of the data is not sufficiently well described to be fully understood. For example, the authors collect data by “monitor[ing] the on-line product catalogs of Licensed Producers each month from June 2016”. There are hundreds and hundreds of LPs. How were these websites monitored? Is this a complete sample of LPs or a highly selected sample?

We concur with the referee. We have revised the methods and results sections to ensure that all data sources used in the manuscript are properly cited.

Re the hundreds and hundreds of LPs: Only a subset of the LPs listed on the Health Canada website sell directly to authorized medical cannabis users and the number of LP vendors was manageable. For instance, in December 2020, there were 71 LPs listed, 38 of which sold to patients. There were 297 varieties of dry cannabis and 98 varieties of cannabis oil. (There were several other dosage forms with small numbers of varieties.)

Very small LPs were excluded. In early 2020, Health Canada created a new class of Licensed Producer called "micro-licence holders". These producers have a maximum limit of 200 square metres of growing surface. In a recently released report,

<https://www.canada.ca/en/health-canada/services/drugs-medication/cannabis/industry-licensees-applicants/licensed-cultivators-processors-sellers.html>

Health Canada notes that the number of micro-licence holders has grown from around 20 at the start of 2020 to 340 as of July 31, 2022, comprising 38% of all federal licence holders.  We excluded them as many do not maintain consumer websites and according to the Health Canada report they account for less than 1% of LP sales.

Re the website monitoring: One of us (MS) visited each LP vendor website at the start of the month and recorded the LP, the strain and the price and potency of each oil or dry cannabis variety on offer. We collected 18,703 observations on dry cannabis products and 6,939 observations on cannabis oil products.

We strived to capture all non-micro LPs that sell to medical cannabis users but some websites required that only authorized cannabis users obtain price information. We make these limitations clear in the text.

Comment R2.3. This is potentially a massive data collection effort that the reader cannot fully understand from the text and may not be provided access to, making it impossible to replicate, to build on, or to examine the subtleties of.

The 25,642 observations on prices, potency and LP of the dry and oil cannabis products that we collected for our study will be included in the Odesi data archive should the manuscript be accepted for publication in PLOS One.

Comment R2.4. The blanket references to unsourceable private administrative data from Health Canada’s Office of Medical Cannabis are even more opaque.

The medical cannabis access regime administrative data we rely on were provided to us directly by our Health Canada contact, Jonathan Fortin. All these data will be included in the Odesi data archive should the manuscript be accepted for publication in PLOS One.

Comment R2.5. The methods section describes a lot of sources, but then the reader struggles to link statistics back to the actual source, especially when these claims are at times extreme. For example, the authors claim the average daily dose of authorized cannabis was 20.6 grams/day in 2013. This is wildly inconsistent with the existing literature that I’m aware of. Unfortunately, I cannot link this statement back to any particular source. The reader is told of many data sources, and then told of many results coming from these sources, but is often not given consistent insight on where certain numbers come from, preventing interpretation of the results.

As noted earlier, we have revised the methods and results sections to better document data sources. These revisions include documenting the source of the 20.6 grams/day figure and the methods used to derive this. The data comes from an affidavit of a Health Canada analyst (Jeannine Ritchot) who testified in the Allard et al federal court trial in Vancouver over the constitutionality of the MMAR. We now reference in the results section the URL of the affidavit

https://johnconroy.com/pdf/Allard-Defendants-Affidavits-Trial/Ritchot-4-Affidavit-1-of-21-pp-1-95.pdf

We rely on data from the table below, which is cut and pasted from Paragraphs 53 and 54 of her Affidavit. This table shows the # individuals who were authorized to possess dry cannabis under the MMAR, by categories of daily authorized amounts (in grams) and year.

We now explain in the methods section that we used these data to calculate, for each year, the weighted average daily dose of medical cannabis. The weights applied to a particular dosage category equals the fraction of individuals using that dosage. The midpoints of the “0 to 2.9” grams per day category was given a midpoint value of 1.5, the midpoint of the “3.0 to 4.9” category was given a midpoint value of 4, and so on. To obtain the annual average, we multiplied the number of individuals using the midpoint #grams per day and summed across the dosage categories. Then we divided this sum by the number of individuals authorized to use cannabis in the year.

Comment R2.6. I agree there is a potentially great resource here if the authors could collate and share 100% of the data from their work to the scientific community, but my reading suggests there is no intention to do so. The work would be much better suited for an outlet focused more on results and less on transparency and process. Alternatively, there is probably enough here for two papers if the authors wanted to split the paper and fully develop the results – perhaps one with the data they can share and one with the data they are unwilling or unable to.

We are agreeable to splitting the paper into two separate papers. One division is a paper that focusses on the LP data we collected plus the HC administrative data (all of which we can share) and another that presents the results of the analysis of the cross-sectional health surveys from 1989-2021. (As noted, we cannot re-publish the source health and drug survey data.).

Splitting the paper in two also makes sense considering the length of the revised manuscript. There are now 10 different figures and two tables and the paper now approaches 30 pages of text.

We are also open to other divisions of the material into two papers.

We defer to the Editor for guidance on this.

Detailed Comments:

Comment R2.7. Fig 2 is an important graph that doesn’t exist this starkly anywhere else in the literature to the best of my knowledge. The source is given as “Health Canada Office of Medical Cannabis”, but the “Office of Medical Cannabis” doesn’t appear in the data section, and I can’t find any website belonging to the OMC that indicates what data might or might not be available? Some discussion of how this data was/is/could be obtained is required.

We now cite the source of these data.

Comment R2.8. The reference [17] attached to the discussion of Figure 2 does not actually provide useful context for the discussion, it just links to a list of LPs but this list seems to be continually updated to reflect changing market conditions and cannot reflect a static list of LPs that the authors actually surveyed in the past.

We remove this citation, as the referee correctly notes that it does not reflect the data we actually collected.

Comment R2.9. Fig 3 is interesting but see earlier comments. Is this using some sort of archived website scraping? Is this a complete sample of LPs? Is this a simple average or weighted by LP size? I don’t think the biggest LP should carry the same weight in this calculation as the 200th-biggest LP.

We agree that we did not adequately describe our methods here. In the revised manuscript we clarify that we used a simple proportion of the number of products on offer in each of the four potency categories (defined by THC and CBD percentages being under or over 10%) by month and cannabis presentation (dry vs oil). We note that, ideally, one would use actual Licensed Producer product sales, but unfortunately these product sales data are proprietary. We also note the potency category share graphs reflect trends in the potency of medical cannabis being used if the number of products in a strength category is proportional to the level of patient demand for all products in the category.

Comment R2.10. The literature should be acknowledged here. My understanding is that other studies that have looked at what’s being sold medically find very different things. In particular the finding that a THC-dominant oil held a commanding market share until 2021 is not what I have read elsewhere.

There are many articles that focus on recreational cannabis varieties but we found just 4 articles that surveyed the presentation (dry vs oil) and/or potency of medical cannabis consumed during the MMPR/ACMPR regimes. All of these are based on patient surveys. These articles confirm that medical cannabis users increasingly are using cannabis oils instead of dried cannabis. But none provide definitive evidence on the potency characteristics of the medical cannabis routinely used. The one article that focussed on the THC-CBD potency of medical cannabis, by Tumati et al (Drugs Aging. 2022 Feb;39(2):153-163), found that cannabis oil was used by 81.0% of seniors (≥ 65 years) that participated in a survey, among whom compositions containing only or mostly cannabidiol (CBD) had been used by 83.6%. This article does question our finding that high THC varieties had a large product share but on the other hand it focuses on just the elderly segment of the patient population.

Comment R2.11. Indeed only 6 of the references are peer-reviewed studies on cannabis. I believe most of what’s being done is novel but there’s limited effort by the authors to place their findings in the literature.

We agree with the criticism. We now situate our findings on interprovincial variation in medical access program participation, medical cannabis product varieties and medical cannabis prices in the extant literature.

Comment R2.12. Fig 4 again interesting, but this is maybe not novel. Statscan maintains good data on prices, probably better data than is presented here.

Re the novelty of our price data: Statistics Canada reports a medicinal cannabis price index by province and by month from January 2019 to October 2022.^[1]^

These price index data are qualitatively different than the data we collect.

First, we collected data on actual prices whereas StatsCan produces an index. Their index can be used to measure price change but not price levels.

Second, we stratified prices by presentation (oil vs dry). Statistics Canada’s medicinal cannabis price index is some weighted average of sub-indices for oil and dry forms. (This information is reported in an on-line document that lists the items that comprise the CPI. <https://www.statcan.gc.ca/en/statistical-programs/document/2301_D68_V1> )

Third, separately by presentation (oil vs dry), we stratify cannabis prices by potency. Statistics Canada does not.

Finally, the Statistics Canada price indices reflect the final consumer price and thus include all federal and provincial general sales taxes and excise taxes. The StatsCan price index begins in January 2019. At this point in time, medical cannabis was subject to tax. Our data collection begins several years earlier. For comparability with our pre-2019 price data, we do not include taxes.

Given these differences, our view is that the 2016-2022 pricing data we collected provides information to the research community that is not available in the Statistics Canada price index.

Re *Statscan maintains good data on prices, probably better data than is presented here*:

Our view is that it is difficult to compare the quality of the two data sources, for several reasons:

First, Statistics Canada does not publicly divulge if or how they stratified by product potency when deriving their index. The closest they come is a note that they rely on “Overall mean imputation, expert judgment” when making quality adjustments to the “health and personal care” component of the CPI. (See Appendix B – General Information on the CPI Major Components of Statistics Canada’s technical guide to the CPI, available at

<https://www150.statcan.gc.ca/n1/pub/62-553-x/62-553-x2019001-eng.pdf>).

Second, Statistics Canada does not publicly divulge information on the procedures used to form a weighted average of sub-indices for oil and dry forms.

Third, it is unclear what data sources Statistics Canada relied on, and the number of products they sampled when constructing these sub-indices.

We do know, however, from paragraph 6.11 of their technical guide that StatsCan does not weight prices based on producer market shares.

**6.11**  In the majority of cases, elementary price indices are based on a sample of prices for one or more goods or services belonging to the elementary product class. The sampled POs receive equal weighting in this elementary calculation, because consumer expenditure weighting information is usually not available at this level.

We do agree that, in general, StatsCan data is of high quality. In the revised manuscript we therefore use the price index data to see if the real cannabis product price decreases that we report are consistent with the national level medicinal cannabis price index decreases reported by Statistics Canada.

Comment R2.13. Fig 4 also claims to be a “mean”, but this is unclear. The data source is given as monthly review of catalogs, but this isn’t a mean because the catalog review doesn’t tell you volumes sold, it’s an average across product lines not by amount sold, and the latter seems to be what we actually want.

We did not adequately describe our methods here. In the revised manuscript we clarify that we used a simple average of the prices of the products on offer (averages were calculated by month, potency and by cannabis presentation, dry vs oil). We note that, ideally, one would weight prices by sales volumes, but LP sales volumes data were not available. This may not be a problem if larger producers tend to sell a larger range of products and thus obtain a larger weight when computing average prices.

Comment R2.14. I think maybe the measurement of oils needs some disclaimers. mL is only relevant if different LPs compound their products at roughly the same % active ingredient, but my understanding is there are significant differences between LPs in the strength of their formulations, so $/mL is possibly not a meaningful statement.

Thanks for this information. We have made this disclaimer when describing these data.

Comment R2.15. The discussion/conclusion section doesn’t clearly make the reader aware of what is novel.

We have more clearly delineated our contribution to the literature in the discussion section of the revised manuscript.

1. These data are online at:

   <https://www150.statcan.gc.ca/t1/tbl1/en/tv.action?pid=1810026301>
