# Supplementary material for: Lessons from 20 years of medical cannabis use in Canada
Source: PLoS One. 2023 Mar 23;18(3):e0271079. doi: 10.1371/journal.pone.0271079 (PMC10035846; doi:10.1371/journal.pone.0271079)
Supplement: Attachment — Submitted filename: response to reviewers r2.docx [file pone.0271079.s003.docx]

Editor, PLOS One

March 8, 2023

Dear Academic Colleague

# Below I indicate how we responded to each of the reviewers’ comments in the second revised version of our research manuscript *Lessons from 20 years of medical cannabis use in Canada*, which we have submitted for publication in PLOS One.

Editor Comments:

Comment E1.

*Based on the comments from one reviewer, the manuscript requires minor revisions: please ensure that the paper, including the abstract, is accessible to an international audience;*

Response: we have ensured that all Canadian institutions and regulations we refer to in the abstract and in the body of the manuscript are explained and thus accessible to an international audience.

Comment E2.

*provide clarification regarding the number of grams authorized; and*

Response: we agree that the cannabis daily dosage authorized by Health Canada under the MMAR regime were very high, perhaps implausibly high. We now clarify that it is unlikely that MMAR program enrollees consumed all the authorized cannabis dosage amounts. We indicate that this seems unlikely given the concerns from law enforcement personnel that cannabis grown under the auspices of the MMAR was being diverted.

Comment E3.

*lastly the manuscript requires a thorough edit to increase clarity, e.g., ensure manuscript is written entirely in the past tense. I look forward to your revised submission.*

Response: we have edited the paper to further increase clarity. As part of these revisions, we ensured that all descriptions of our methods are written in the past tense.

Reviewer #1 comments

Comment R1.

*The revised manuscript includes additional information requested by the other reviewer and me. The authors’ responses to our question and suggestions were thoughtful. I continue to think the information on the evolution of medical cannabis is Canada presented in the manuscript will be useful to the field.*

*The authors do not run statistical models assessing statistical significance of associations or estimating magnitude of “effects” of policy changes. I think that is fine, although there are place in the paper where the authors should make edits to be more cautious about causal inferences.*

# Response: we removed any statements that implied that a specific policy change exerted a causal impact on the outcome variables studied in this paper.

Comment R2.

*I had requested the authors make sure their paper was accessible to an international audience. The authors added some clarification on how the system of physician Colleges works in setting province-specific policies into the main body of the paper. It would help readers if the Abstract was written in a way that made it clear what Health Canada and physician Colleges are.*

Response: we have ensured that all Canadian institutions and regulations we refer to in the abstract and in the body of the manuscript are explained and thus accessible to an international audience.

Comment R3.

*Both the other reviewer and I reacted to information reported on average daily doses of cannabis. The response letter provided some reasons why the average authorized dose under MMAR would have been so high. My takeaway from the authors response is that the number of grams authorized under MMAR, the average number of grams supplied under MMPR, and the actual number of grams per day consumed by medical cannabis users are very different things. It would help the reader if the authors added some clarifying information to the text of the manuscript. If they don’t, many readers, thinking in terms of actual grams consumed, are going to think that the high numbers are due to typographical or data coding errors.*

Response: we agree that the cannabis daily dosage authorized by Health Canada under the MMAR regime were very high. We now clarify that it is unlikely that MMAR program enrollees consumed all the authorized cannabis dosage amounts. We indicate that this seems unlikely given the concerns from law enforcement personnel that cannabis grown under the auspices of the MMAR was being diverted.

Comment R4.

*In general, the writing quality of the paper is fine in terms of clarity. That said, this paper presents a lot of information and, if you are not Canadian, a lot of that information is new and confusing. The content of the paper also does not neatly fit into the conventional format of journal article. For example, should history of medical cannabis policy be in the Results section of the Abstract?*

Response: we agree that the content of the paper does not lend itself well to the standard intro/methods/results/discussion format. That is why we felt it was warranted to add some additional sections. Regarding the history of medical cannabis policy being in the Results section of the Abstract, we felt it important to provide some information on the regulatory policy changes to highlight the research questions and results.

Comment R5.

*The structure of the Abstract and the body of the manuscript do not match.*

Response: we agree – the Abstract now highlights the questions asked in the paper and the key results.

Comment R6.

*The paper could be published in close to its current form, but I believe the authors would be much happier with their paper, and it would receive a much wider audience, if it received a thorough edit to increase clarity, eliminate some typographical and grammatical errors, increase precision of word choice, and, in general, make the paper more reader friendly. One recommendation I have is that the paper be written almost entirely in the past tense. There are several places where the authors slip into the present tense, which tends to impart stronger inferences than the past tense. The strength of inferences is general issue with this paper. The manuscript is primarily descriptive. I would caution against make statement that imply specific policy changes “caused...”*

Response: we have edited the paper to further increase clarity. As part of these revisions, we ensured that all descriptions of our methods are written in the past tense. As noted above, we removed any statements that implied that a specific policy change exerted a causal impact on the outcome variables studied in this paper.
